# A Rare Case of Calcified Simple Mesenteric Cyst in a 70-Year-Old Woman

**DOI:** 10.1155/2022/8692421

**Published:** 2022-11-02

**Authors:** Alazar Berhe Aregawi, Alemwosen Teklehaimanot Alem

**Affiliations:** ^1^Department of Surgery, Hawassa University Comprehensive Specialized Hospital, Hawassa University, Hawassa, Ethiopia; ^2^Department of Pathology, Hawassa University Comprehensive Specialized Hospital, Hawassa University, Hawassa, Ethiopia

## Abstract

Mesenteric cysts are one of the rare causes of intra-abdominal masses. They account for 1 in 100,000 cases in adults and 1 in 20,000 cases in children. Mesenteric cysts are commonly found in the small intestine, up to 60% of cases, and occasionally in the colon. The clinical presentation of patients with mesenteric cysts is so variable and nonspecific. It ranges from being asymptomatic to features of acute abdomen very rarely. Surgery is the treatment of choice. Complete excision with negative margins plays a curative role in avoiding the risk of recurrence as well. Here, we present the case of a 70-year-old woman who came to Hawassa University Comprehensive Specialized Hospital with abdominal swelling for a 5-month duration. She had an abdominal ultrasound, which suggested a calcified mesenteric cyst with internal hemorrhage. The patient was taken to the OR with the impression of an intra-abdominal mass for exploratory laparotomy. The mass was completely excised and was subjected to pathology, which turned out to be a calcified simple mesenteric cyst, and the purpose of this case report is to alert physicians that although the preoperative diagnosis of mesenteric cysts is difficult, it should be considered in the differential diagnosis of a patient presenting with an intra-abdominal mass.

## 1. Introduction

Mesenteric cysts are one of the rare causes of intra-abdominal masses. They account for 1 in 100,000 cases in adults and 1 in 20,000 cases in children [1–3]. Nearly 33% of cases occur in children [3]. Mesenteric cysts are commonly found in the small intestine, up to 60% of cases, and occasionally in the colon. They can arise from anywhere starting from the duodenum up to the rectum, but the ileal mesentery is the common site [1–7]. So far, less than 1000 cases have been reported in the literature, and the first case was reported in 1507 by Benevieni [2, 5, 6, 8].

Mesenteric cysts commonly occur in the Caucasian population in their forties, and females are slightly more affected [2, 6]. Though there are many theories, the exact etiology and mechanism of formation of mesenteric cysts are unclear [2]. Mesenteric cysts can be single or multiple, but majority are solitary cysts [2, 6]. The content of the cysts can be serous, chylous, or dark brown, in case there is hemorrhage [6].

The clinical presentation of patients with mesenteric cysts is so variable and nonspecific. It ranges from being asymptomatic to features of acute abdomen very rarely [2, 3, 8]. The presentations can be categorized in to three groups: (1) those with nonspecific abdominal features, (2) those diagnosed incidentally following abdominal imaging, and (3) those with features of acute abdomen [6]. Development of symptoms depends on the size of the cyst, the location, and rarely whether there are complications like intestinal obstruction, volvulus, hemorrhage, or peritonitis [1, 2, 6].

According to the most widely used classification of mesenteric cysts by Miljković et al. [9], which takes account histopathological features and cells of origin, six groups of mesenteric cysts are recognized: lymphatic, mesothelial, intestinal/enteric/, urogenital, dermoid cysts, and nonpancreatic pseudocysts [2, 9, 10]. The etiology of mesenteric cysts is unclear, but the proposed mechanisms include developmental abnormalities, trauma, and mechanical obstruction to lymphatic channels, and they are not mutually exclusive [3, 6].

Preoperative diagnosis of mesenteric cysts is often difficult, but it should be considered in the differential diagnosis of a patient presenting with an intra-abdominal mass [1, 2]. A number of therapeutic approaches have been suggested for treating mesenteric cysts depending on the size and location of the cyst and whether the patient is symptomatic or not. Few authors propose a wait-and-see attitude in asymptomatic cases, and most authors recommend radical cyst removal, given the risk of developing complications like malignancy due to the cyst [11]. The other less invasive therapeutic approach which is recently being used is interventional radiology, especially for percutaneous drainage procedures with or without ethanol ablation. However, this procedure carries the risk of leaving a malignant or potentially malignant lesion in situ and carries the risk of subsequent recurrence [12]. Thus, surgery is the treatment of choice. Complete excision with negative margins plays a curative role avoiding the risk of recurrence, provides a definitive histological diagnosis, and prevents the development of possible complications as well [2, 13]. As the risk of malignant transformation in mesenteric cysts reaches up to 3% and even higher, when there are solid components, surgical excision is recommended [1, 2].

“Once diagnosed, all mesenteric cysts should be resected to avoid complications, recurrence, malignant transformation, and possible complications (hemorrhage, torsion, obstruction, traumatic rupture, and infection). Internal drainage may be an option when there is a possibility of short bowel syndrome. In selected cases, laparoscopic approach can be used” [8].

## 2. Case Presentation

We present a case of a 70-year-old woman who came to Hawassa University Comprehensive Specialized Hospital with abdominal swelling for a 5-month duration. She claimed to have a dragging sensation occasionally. She had no abdominal pain. She had no vomiting or bowel habit changes. She had no anorexia. She had no weight loss. She had no constitutional symptoms of malignancy.

Her past medical history was unremarkable.

### 2.1. Physical Examination

#### 2.1.1. On General Appearance

She looked comfortable. All the vital signs were within the normal range; on abdominal examination, there was a grossly visible mass in the area of the umbilicus and epigastric area, and it was firm, nontender, and slightly mobile; there was no sign of fluid collection. Digital rectal examination was normal.

### 2.2. Investigation

#### 2.2.1. On Serology

- CBC − WBC = 6.1 × 10^3^/*μ*L; granulocytes = 70.1%; lymphocytes = 21.1%
- Hgb = 12.9; platelet = 367 × 10^3^
- Bg&Rh = o+
- FBS = 105 mg/dL
- BUN = 16
- Creatinine = 0.8
- ALP = 77
- AST = 24
- ALT = 35

#### 2.2.2. On Imagings

**Figure 1:**
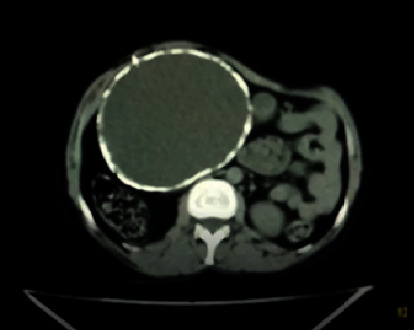
Abdominal CT scan.

- CXR-normal
- *Abdominal ultrasound*. There was a mid-abdomen region, a large well-defined cystic mass with multiple wall calcifications, internal echo debris, and fluid levels. There were also multiple wall calcifications. It measured 13.9^∗^13.6 cm. No vascular flow was detected. No soft tissue components were noted. The DDx on abdominal ultrasound was a calcified mesenteric cyst with an internal hemorrhage and recommended abdominal CT, and it was done (Figure 1).
- *On the CT scan*. There was no evidence of malignancy, and there was no invasion of adjacent organs or major vessels

**Figure 2:**
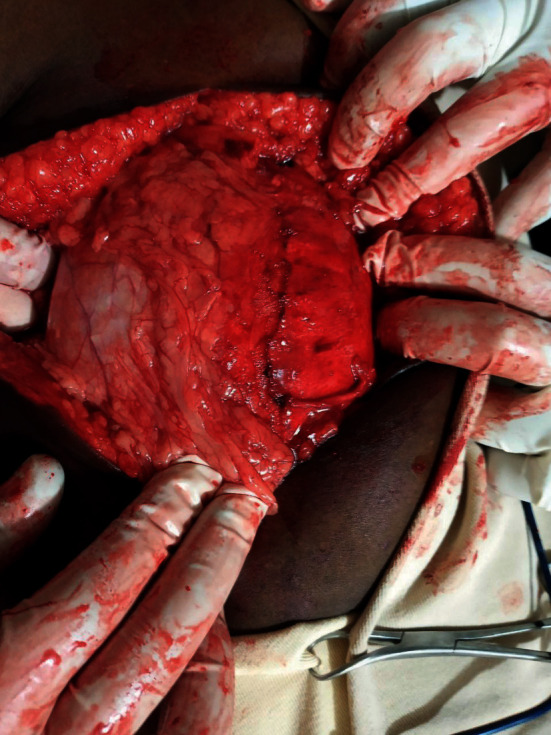
The mass with its abdominal wall attachment.

**Figure 3:**
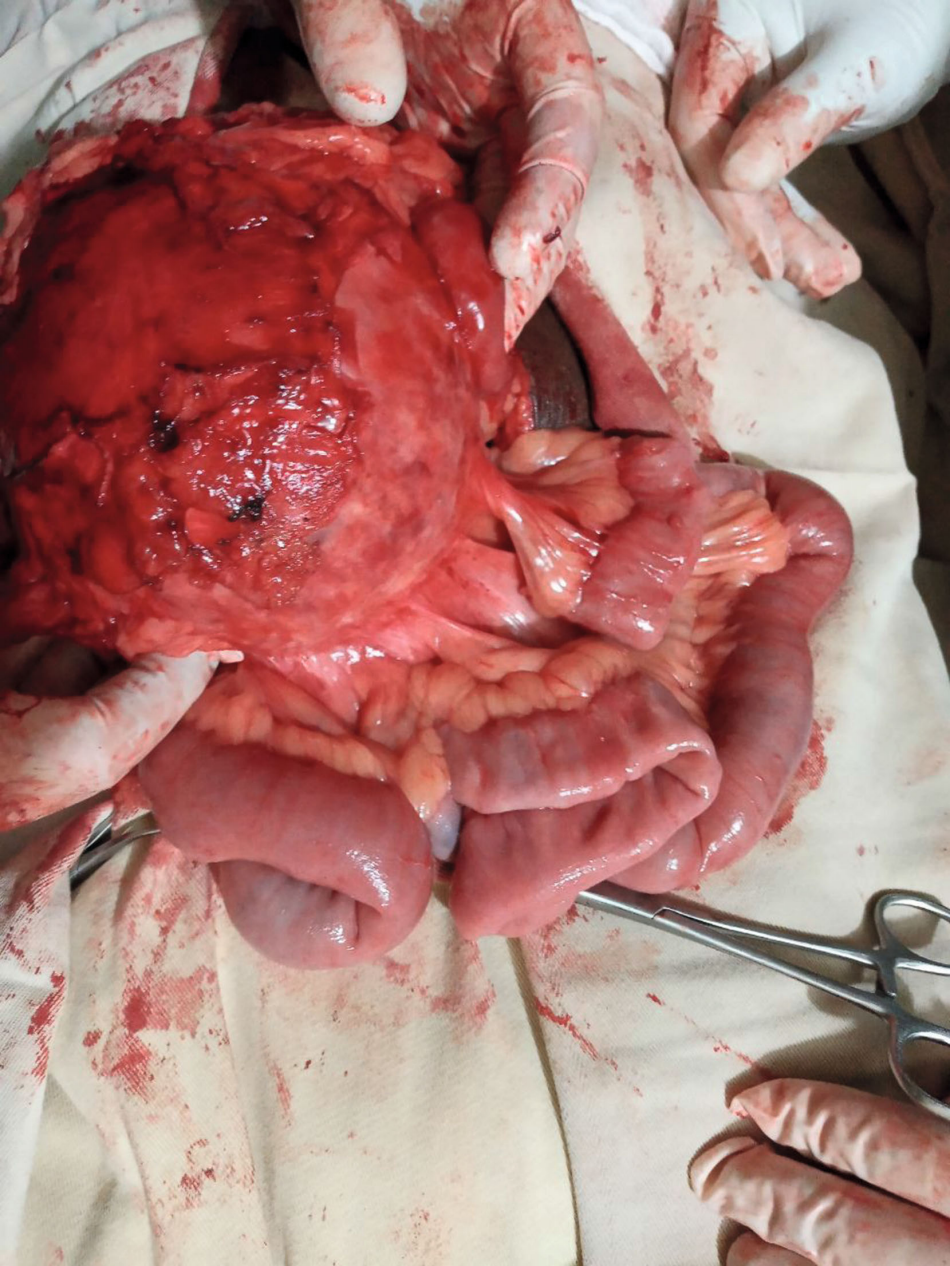
Cyst arising from the small bowel mesentery.

**Figure 4:**
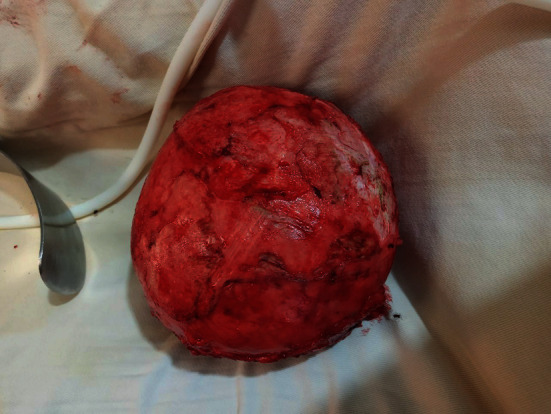
The excised calcified cyst.

**Figure 5:**
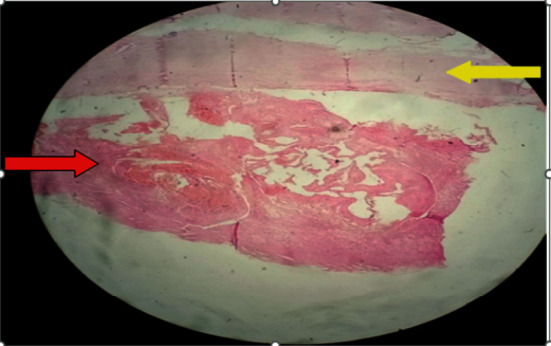
Low-power photomicrograph revealing fibrotic cyst wall (yellow arrow) and hyalinized pinkish cyst content (red arrow).

**Figure 6:**
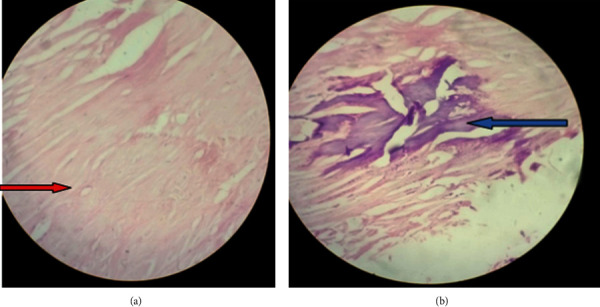
(a, b) High-power photomicrograph revealing an acellular fibrotic cyst wall (red arrow) and focus of calcified cyst wall (blue arrow).

**Figure 7:**
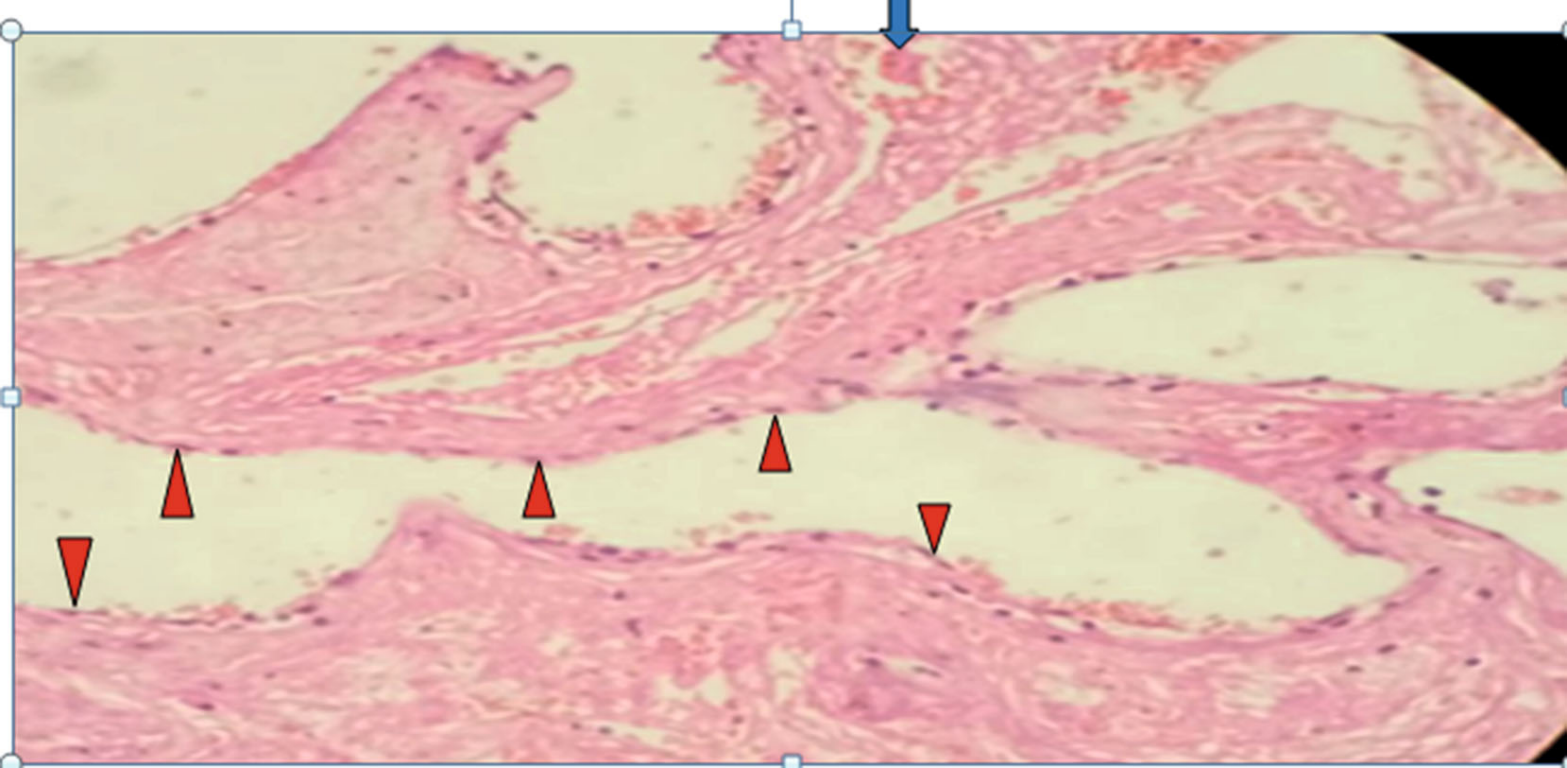
High-power photomicrograph revealing foci of inner cyst wall lined by a single layer of bland flattened epithelial cells, likely mesothelial cells (red arrowheads) and foci of hemorrhage (blue arrow).

The patient was taken to the operating theater with the impression of an intra-abdominal mass for exploratory laparotomy. The abdomen entered through a midline vertical incision. Upon opening the linea alba, the mass was found to be adherent to the anterior abdominal wall, and it was thick walled (Figure 2). The mass was gently released from the anterior abdominal wall, and it was found to arise from the jejunal mesentery. The greater omentum had wrapped around much of the surface of the mass. The mass was completely excised without sacrificing the mesenteric vessels and without rupture (Figures 3 and 4). It measured about 20 × 18 × 14 cm. There were no regional lymph nodes and no ascites. The rest of the bowel, pancreas, and liver looked normal. Finally, the abdomen closed in layers after the count was correct. There was no intraoperative incident. The mass was subjected to histopathology examination (Figures 5–7). The patient had an uneventful postoperative course and was discharged on the 4th postoperative day.

## 3. Discussion

Simple mesenteric cysts are rare, and calcified ones are rarer conditions, and not more than 1000 cases have been reported so far in the literature [4]. They do have variable sizes ranging from a few centimeters to large sizes with an estimated volume of 5 liters [10]. Our patient's cyst measures about 20 × 18 × 14 cm, which is relatively big.

There are no symptoms or signs which are considered pathognomonic for mesenteric cysts [6, 8]. Cysts, which are typically asymptomatic, can be identified during regular imaging tests. Chronic abdominal pain, a palpable mass, abdominal distension, nausea, vomiting, constipation, or diarrhea may be the presentations of symptomatic cases. In such cases, there is debate whether the therapeutic approach should be radiologic or surgical. Alternatively, patients may present to the emergency department with acute GI symptoms such as abdominal pain due to a complete bowel obstruction, peritonitis, or volvulus from the outset requiring emergency surgery [13].

Commonly, mesenteric cysts are asymptomatic with typical transverse mobility and around the axis of the mesentery, but occasionally, patients may present with nonspecific symptoms like abdominal pain or nausea [5, 10]. Our patient had a dragging sensation, and the mass did not have the typical mobility. The size of the cyst is not a mere factor for the presence of symptoms. It is the location of the cyst in the abdomen that is the most determining factor and whether an individual develops symptoms or not. External pressure is the principal mechanism, whereby symptoms develop [10]. Mesenteric cysts can cause an acute abdomen in the pediatric population [1].

According to 22 patients who were operated between January 2010 and January 2019 after the diagnosis of mesenteric cysts in the General Surgery Clinics of Şanlıurfa Training and Research Hospital, and Mehmet Akif Inan Training and Research Hospital, 12 cases presented with abdominal pain, 3 cases with nausea and vomiting, 2 cases with an abdominal distension, 2 cases with abdominal mass, and in the other 3 diagnosis were made incidentally during abdominal imaging [1]. And according to a case series by Arq and Cir, out of the 18 cases of mesenteric cysts 72% presented with abdominal pain and mass, only one case presented with a feature of acute abdomen [8]. Our case presented with abdominal swelling.

Regarding sex predilection of mesenteric cysts, previous studies suggested that men were more affected than women, but more recent studies show otherwise. The female-to-male ratio is about 2 : 1 [5]. This was demonstrated as well in the case series of 22 cases of mesenteric cysts, where 63.6% cases were females [1]. Our case is as well a female patient.

Although it is the first imaging that is done, ultrasound is not the first choice of imaging modality for intestinal and mesenteric masses as it lacks precision in detailing the relationship of the mass with the surrounding tissues and viscera [10]. Abdominal CT is recommended for a detailed evaluation. CT is very useful for localization, preoperative planning, and assessing the relation of the cyst with adjacent organs [1, 10]. MRI has had a better yield of cyst evaluation [2]. In general, mesenteric cysts have benign features on imaging [1, 3]. Both abdominal ultrasonography and CT were performed in this study, which suggested a calcified mesenteric cyst with no features of malignancy.

Concerning the treatment of mesenteric cyst, complete excision with or without bowel resection, depending on the involvement of the mesenteric vessels or the bowel itself, is the treatment of choice [5, 6]. Simple aspiration or marsupialization may be considered when short bowel syndrome is anticipated, but these procedures are generally unadvised due to the risk of infection and frequent recurrence [5]. Percutaneous ablation with ethanol is another option mentioned in the literature. It is recommended for cysts considered benign, have a smooth and thin wall, and are unilocular [14]. Few studies recommend that active follow-up is enough for asymptomatic cases, but many authors recommend radical removal of the mass even in asymptomatic patients. Even authors who consider the possibility of spontaneous regression advocate complete surgical excision as the best option [10]. Our case had complete surgical excision.

The choice of surgery depends on the location of the cyst, its size, and whether adjacent organs are involved or not. According to Yavuz, who analyzed 22 cases of mesenteric cysts managed surgically between January 2010 and January 2019, 11 cases had enucleation, 4 cases had segmental small bowel resection and anastomosis, 3 cases had laparoscopic cyst excision, 2 cases had left hemicolectomy, 1 case had right hemicolectomy, and 1 case had appendectomy and cystectomy [1]. Given that the pathology is rare, statistics are lacking in the risk of malignancy in this benign condition. “There are few reports of malignant mesenteric cysts, usually low-grade sarcomas. Kurtz et al. reviewed 162 cases and found only 3% of malignant transformation, all in adults” [8]. Three cases of adenocarcinomas have been reported in the literature [6]. Therefore, complete surgical excision is considered the best option. However, the other important parameter to be considered regarding the option of management is the location of the cyst, even in asymptomatic patients [10].

Open surgery is considered the best modality. Despite the well-known benefits of laparoscopy over open surgery, there are very few cases of laparoscopic excision of mesenteric cyst. It is said that advanced skill must do it laparoscopically [2, 10]. Our case was managed with an open procedure. Other treatment modalities are cyst aspiration and marsupialization, which are highly associated with recurrence and infection; hence, they are not routinely recommended [2, 6, 15].

Where the patient is a poor surgical candidate, cyst aspiration was attempted in some cases, and in about 1/3^rd^ of the cases, the cysts recurred very rapidly [10]. Drainage later followed by surgery has been used as a temporary measure to relief symptoms in some cases [10].

Mesenteric cysts and cystic lymphangiomas are two rare entities that have almost similar clinical and radiological appearances, but they do differ in histological basis [10]. The presence of small-lymphoid aggregates in the cyst wall is an important tool to distinguish lymphangiomas from simple mesenteric cysts [10]. Mesenteric lymphangiomas are less common than mesenteric cysts and usually occur during childhood [1]. It is crucial to determine whether a mesenteric cyst is a lymphangioma or not since it can occasionally become aggressive. Although cystic lymphangioma is usually benign, occasionally, it can invade adjacent tissues and organs [10].

As most mesenteric cysts are benign, they have an excellent prognosis following a complete surgical excision with little risk of recurrence [2]. Our case was histomorphologically confirmed as a simple mesenteric cyst (calcified) and had no features of recurrence after one year.
